# Dementia-Friendly Design: A Set of Design Criteria and Design Typologies Supporting Wayfinding

**DOI:** 10.1177/19375867211043546

**Published:** 2021-09-14

**Authors:** L. P. G. van Buuren, M. Mohammadi

**Affiliations:** 1 Department of Built Environment, 3169 Eindhoven University of Technology, the Netherlands

**Keywords:** comparative floorplan analysis, dementia, inpatient care facilities, typological floor plan, design criteria, wayfinding

## Abstract

**Objectives, purpose, or aim:**

This study aims to gain insights into the implementation of theoretical knowledge on dementia-friendly design into practice to (1) identify key design criteria stimulating spatial orientation and wayfinding for seniors with dementia and (2) determine the optimal design for this purpose.

**Background:**

Spatial orientation problems of seniors with dementia can be counteracted by the design of the physical environment of inpatient care facilities. Research has been conducted about design features supporting wayfinding skills for this target group, however, not on their implementation.

**Methods:**

Fourteen floor plans of the living group of built projects have been evaluated on 14 design criteria supporting wayfinding skills for the target group and measurable in floor plans by the performance of a comparative floorplan analysis and multicriteria assessment.

**Results:**

Although one third of the evaluated design criteria are properly implemented, all floor plans of the selected projects had some gaps in fulfilling all design criteria. Five typological floor plans—based on the circulation systems of the cases—were distinguished: one straight corridor structured by two walls, one corridor with corners, two corridors separated from each other by the living room, a continuous loop corridor, and a corridor framed by a wall and interior elements (e.g., cabinets). The majority of the cases was based on a linear system with one straight corridor.

**Conclusions:**

Based on this study, three of the five discovered typological floor plans work well for stimulating wayfinding. Furthermore, special attention need to be given to the configuration of the floor plans, shape, and daylight in the corridor.

## Dementia, Wayfinding, and the Spatial Environment

The number of people with dementia in the Netherlands is rising, from 270,000 inhabitants in 2017 to 520,000 inhabitants in 2040 (Alzheimer Nederland, 2020). Dementia is a general term for a gradual decline in mental ability that is severe enough to interfere with daily life (Verhaest, 2008). Seniors with dementia in a late stage are unable to live at home anymore and have to move to an inpatient care facility (nursing home; Den Draak et al., 2016). The symptom of a decline in spatial orientation occurs already in early stages (Jonker et al., 2009).

Arthur and Passini (1992) defined spatial orientation as “the process of devising an adequate cognitive map of a setting along with the ability to situate oneself within that representation” (p23). This definition represents a static relationship between the user and the space they occupy, while wayfinding implies a dynamic interaction between the spatial environment and its occupant. This relationship is defined by Arthur and Passini (1992) as “spatial problem solving comprising the following processes: decision making, decision executing, and information processing” (p25).

As wayfinding is a matter of the execution of wayfinding decisions (Passini, 1996), people need to understand clearly their position in space and the position of their destination (Brush & Calkins, 2008). Regrettably, seniors with dementia are (in the process of) losing that ability.

Karol and Smith (2019) state that the physical environment impacts residents by empowering them to execute daily activities and influences residents’ feelings by establishing an atmosphere using features like colors, materials, lighting, and shape. In line with the first impact, architecture and design features related to people’s circulation—such as spatial layout, furnishing, signage, colors, and graphic displays—can support wayfinding abilities in a two- and three-dimensional level (Marquardt, 2011; Passini, 1996). However, poor and inadequate architectural features could cause wayfinding difficulties (Marquardt, 2011; Passini, 1996).

## The Need for Designing Dementia-Friendly Architecture

The environmental docility hypothesis (EDH) is used as the theoretical framework in this study. The EDH argues that people with restrictions on their health or cognitive ability are more dependent on their environment as it is harder for them to adapt the environment to their needs (Lawton & Simon, 1968). Marquardt and Schmieg (2009) state that “this implies that people with dementia have lesser capacity to regulate the environmental factors, so their environment should be designed in such a way that it meets with their specific needs” (p333).In this study, these facilities need to be designed in such a way that spatial orientation and wayfinding skills of seniors with dementia are supported. It is, therefore, important to know how these buildings should be designed. Several literature sources have focused on design features to support wayfinding for seniors with dementia (e.g., Day et al., 2000; Marquardt, 2011; Passini et al., 2000; Zeisel et al., 2003). However, a study into implementing these design criteria in actual practice has not been conducted yet. Therefore, 14 floorplan layouts of existing inpatient care facilities in the Netherlands are evaluated in this study. 14 design criteria supporting wayfinding and spatial orientation for seniors with dementia form the foundation of the evaluation, which will add practical knowledge to the EDH database on how this kind of buildings should be designed to meet residents’ needs.

***Several literature sources have focused on design features to support wayfinding for seniors with dementia.***

***However, a study into implementing these design criteria in actual practice has not been conducted yet.***

This study focuses on designing inpatient care facilities for seniors with dementia to support their spatial orientation and wayfinding skills. The aim of the study is to gain insights into the implementation of theoretical knowledge on dementia-friendly design into daily practice on two levels: (1) which and how design criteria are implemented in daily practice and (2) which design layouts (spatial configuration) of the cases support wayfinding. The study is based on a comparative floorplan analysis (CFA) and a multicriteria assessment (MCA) conducted on floorplan layouts of the selected existing inpatient care facilities. The originality of the study lies in evaluating the performance and effects of the design of inpatient care facilities on the wayfinding of residents with dementia in real-life practice.

To improve inpatient care facilities, this study focuses on the crucial places for the resident: the entrance, the corridor, the living room, the bathroom, and the individual room of the resident (Nillesen & Optiz, 2013; van Liempd et al., 2009). The entrance of an inpatient care facility occurs on different levels, namely, of the building complex, living group, and the entrance of the individual room. The transition space between “outside” and “inside” is a differentiation in the degree of privacy. The corridor is often a connecting element between spaces in the inpatient care facilities. However, from an archetypal point of view, a house has no corridors. Corridors in an inpatient care facility are often large spaces, and the residents do not recognize the space as a corridor. The living room is the collective space in the dwelling of the living group where the residents can come together to undertake activities and to eat and contains the following facilities: kitchen, living room, dining area, and a space for activities (Elmstahl et al., 1997; Zeisel et al., 2003). The individual rooms of the residents are the space where the resident can retract (alone or with visitors) and sleep. Lastly, the bathroom has a toilet, a shower, and a sink and is the most private area.

### Method

### Cases: Floorplan Layouts of Existing Inpatient Care Facilities

Through a CFA, floorplan layouts of the “living group” (in Dutch: woongroep) of existing inpatient residential care facilities for seniors with dementia in the Netherlands were evaluated on design criteria (see “Design Criteria” section) supporting wayfinding. The selection of these facilities was based on the following selection criteria. First, cases were selected which (1) won, were nominated for, or recognized in Hedy d’Ancona Award (2010, 2012, 2014, and 2016) and the “International Building Award” 2016 (respectively, a Dutch and an international award for healthcare architecture); (2) were published on the Dutch recognized platforms for design and development of nursing homes, such as the website of “De Architect” (The Architect) and the website of the Dutch branch organization of care entrepreneurs specialized in care homes, Aedes-Actiz; or (3) mentioned on the list of Top 10 housing options Aedes-Actiz. The first step resulted in a list of 21 cases. Involved stakeholders and (urban) context were two important variables. Therefore, the selection of cases was narrowed down via a (1a) variety of architectural firms to create a differentiation in possible architectural translations of inpatient care facilities, (1b) a variety of healthcare organizations to have a differentiation in care processes, and (2) a variety in urban and rural locations to have a differentiation in context. This part resulted in a list of 14 cases of inpatient care facilities designed by 13 different architectural firms and 13 different healthcare organizations. Table 1 shows an overview of the cases. Additional information and two drawings represent the floorplan layout on the building complex level and the living group level (the same type of spaces in each floorplan layout is filled with the same color). In this article, the floorplan layouts of the cases will be called “C([A])” instead of their official names for readability matters.

**Table 1.** Overview of the 14 Cases in the Netherlands for the Assessment.

Basic Information: Name, Location, Year of Completion, Architect, and Care Organization	Building Complex	Floor Plan of Living Group	# Living Groups Per Floor	# Residents Per Living Group	Principle of Circulation System	Length of the Longest Route From the Individual Room to the Living Room	Width of the Corridor at the Smallest Part	Principle of Sanitary Room
(A)BOSWIJK Vught, 2013 EGM Architecten Van Neynselgroep	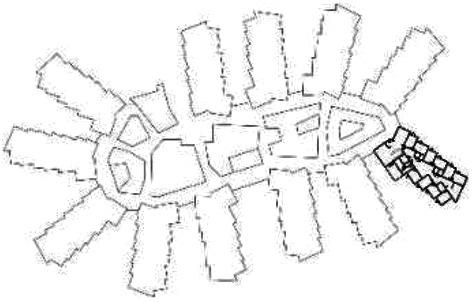	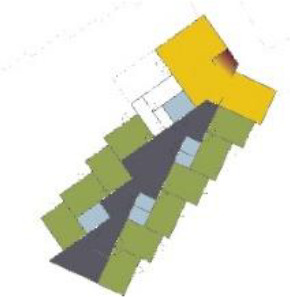	12	10	Linear	27 m (88.58 feet)	2,122 m (6.96 feet)	Shared with >2
(B)DE KEYZER Amstedam, 2011 Frantzen et al architecten Amsta	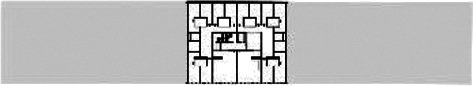	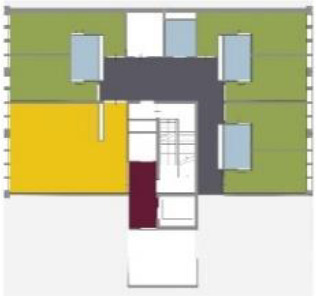	2	6	Linear	16 m (52.49 feet)	1,354 m (4.44 feet)	Shared with 2
(C)DE KOEKOEK Veenendaal, 2014 Studio ID+ QuaRijn	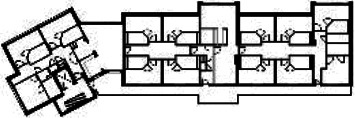	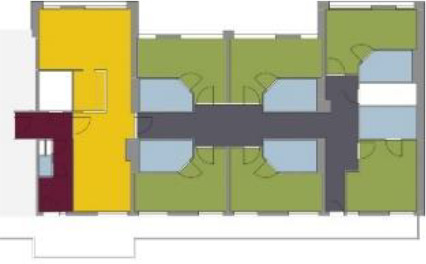	2	6	Linear	18 m (59.06 feet)	1,785 m (5.86 feet)	Individual
(D)DE RIETVINCK Amsterdam, 2009 Marc Posman Architecten Osira groep	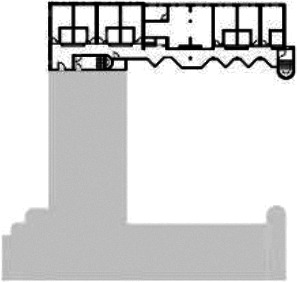	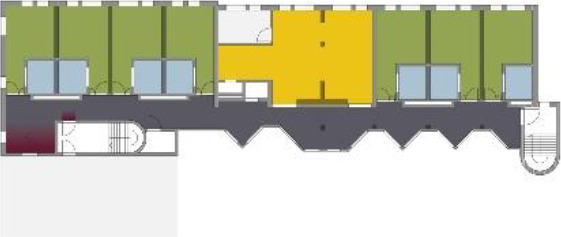	3	7	Linear	23 m (75.46 feet)	1,346 m (4.42 feet)	Individual
(E)DE SCHIPHORST Meppel, 2013 B+O Architecten Zorgcombinatie Noorderboog & Vanboeijen	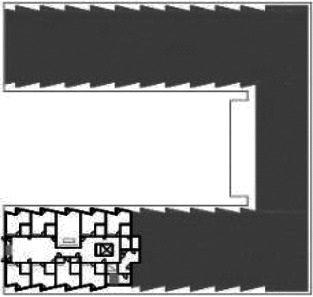	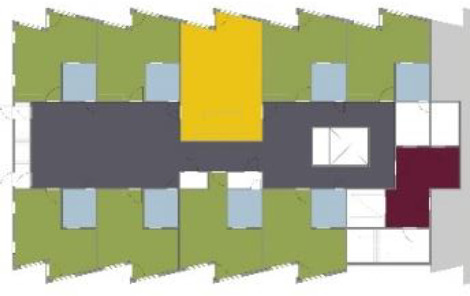	4	8	Linear	15 m (49.21 feet)	1,661 m (5.49 feet)	Individual
(F)DE ZEVEN BRONNEN Maastricht, 2014 Verheij Architecten Heem Wonen	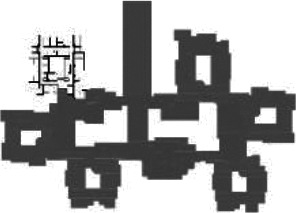	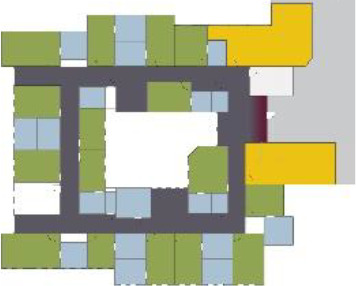	7	16	Circular	41 m (134.51 feet)	2,039 m (6.69 feet)	Individual
(G.A)HEIVELD (A)Landgraaf, 2015 iNeX Architecten Heem Wonen	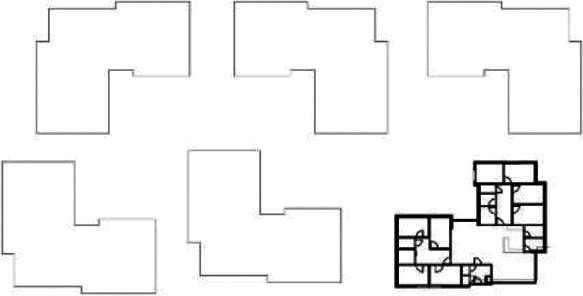	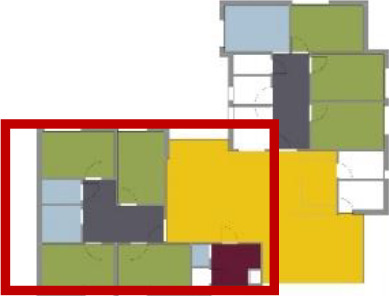	6	7	Two corridors	8 m (26.25 feet)	2,085 m (6.84 feet)	Shared with >2
(G.B)HEIVELD (B)Landgraaf, 2015 iNeX Architecten Heem Wonen	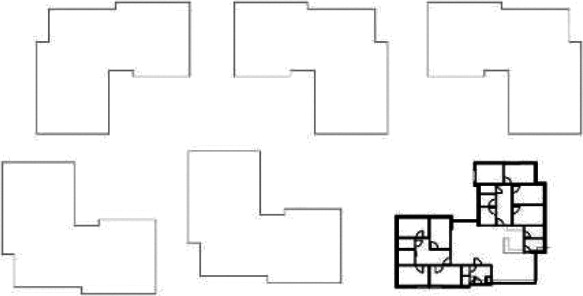	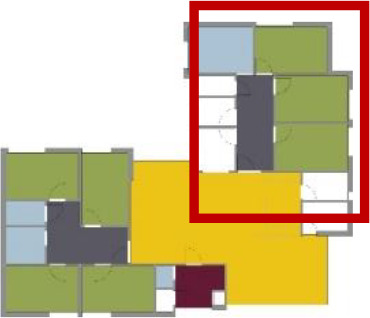	6	7	Two corridors	8 m (26.25 feet)	2,085 m (6.84 feet)	Shared with >2
(H.A)HOGEWEYK (A)Weesp, 2009 Molenaar & Bos & van Dillen Architecten Vivium Zorggroep	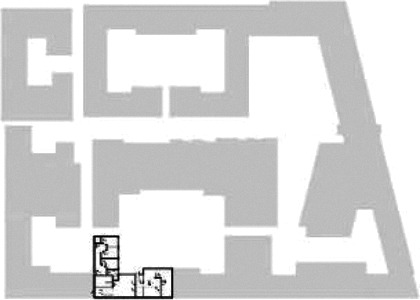	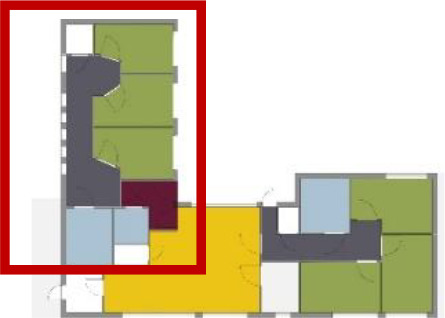	16	6	Two corridors	15 m (49.21 feet)	1,599 m (5.25 feet)	Shared with >2
(H.B)HOGEWEYK (B)Weesp, 2009 Molenaar & Bos & van Dillen Architecten Vivium Zorggroep	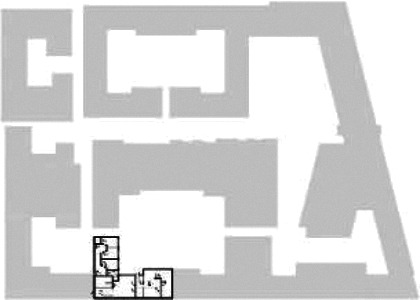	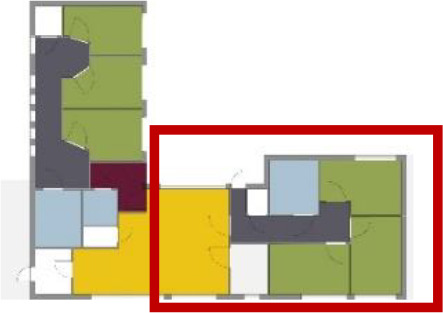	16	6	Two corridors	15 m (49.21 feet)	1,599 m (5.25 feet)	Shared with >2
(I.A)ISSELWAERDE (A)Ijsselstein, 2013 EGM Architecten AxionContinu	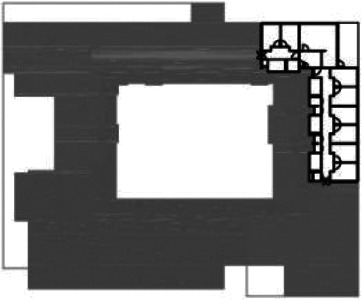	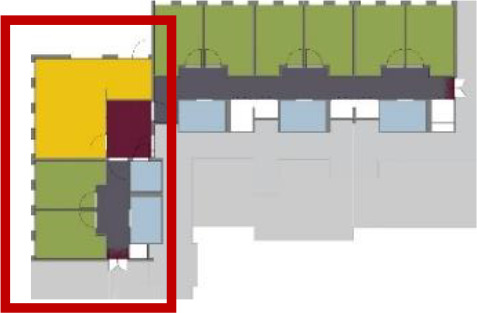	4	8	Two corridors	27 m (88.58 feet)	1,476 m (4.84 feet)	Shared with 2
(I.B)ISSELWAERDE (B)Ijsselstein, 2013 EGM Architecten AxionContinu	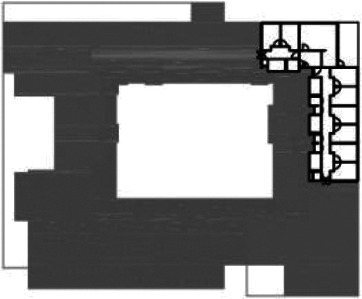	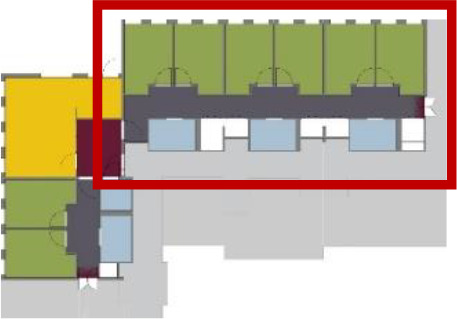	4	8	Two corridors	27 m (88.58 feet)	1,476 m (4.84 feet)	Shared with 2
(J)JULIANA Nijmegen, 2016 FAME Planontwikkeling ZZG Zorggroep	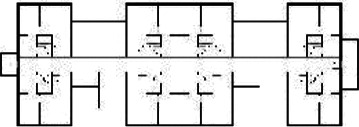	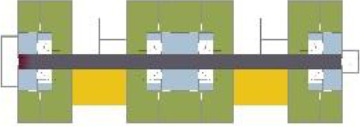	1	8	Linear	15 m (49.21 feet)	2,000 m (6.56 feet)	Individual
(K)KULTURHUS LITSERBORG Den Dungen, 2015 Architecten aan de Maas Brabant Wonen Vivent	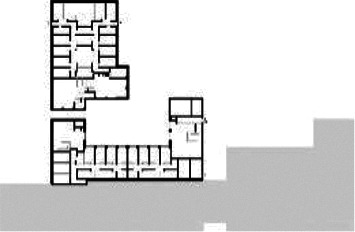	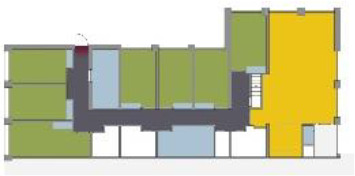	4	7	Linear	21 m (68.90 feet)	1,399 m (4.59 feet)	Shared with >2
(L)ST. ELISABETH Amersfoort, 2016 Ebbens Architecten Beweging 3.0	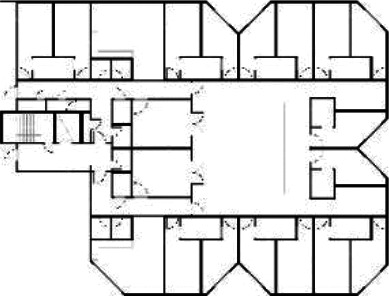	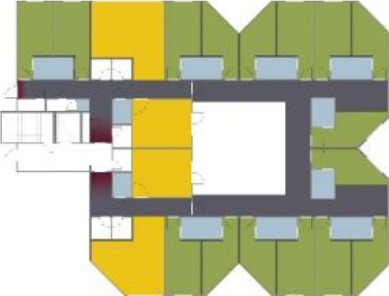	1	16	Circular	20 m (65.62 feet)	1,552 m (5.09 feet)	Shared with 2
(M)‘T LOUG Delfzijl, 2012 Wiegerinck Stichting De Hoven	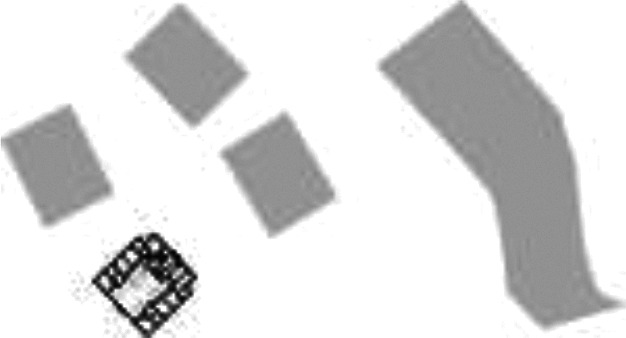	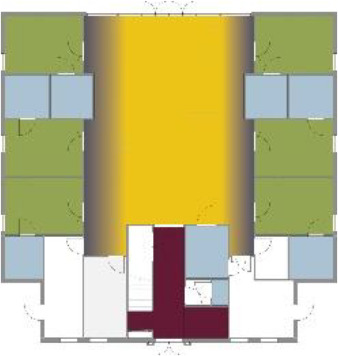	4	6	Special	8 m (26.25 feet)	1,494 m (4.90 feet)	Individual
(N)WIJERODE Heerlen, 2014 DMV Architecten Mondriaan	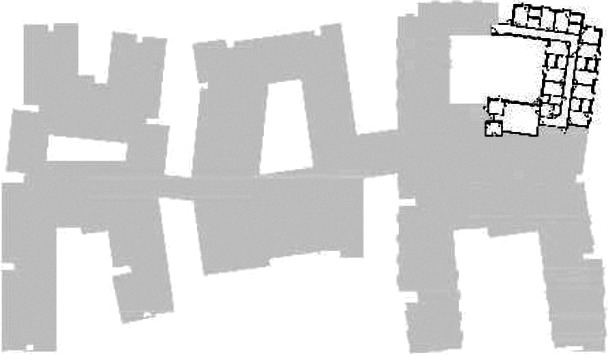	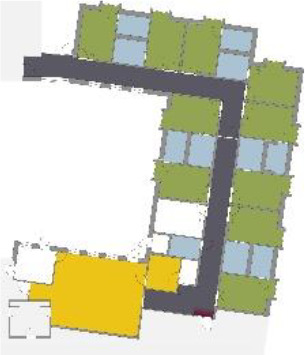	8	10	Linear	43 m (141.08)	1,875 m (6.15 feet)	Individual
Legend:	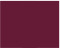 Entrance	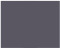 Corridor	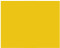 Living room		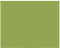 Individual room		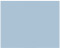 Bathroom

The floorplan layouts of the selected cases are distinct in terms of their characteristics. They are spread throughout the Netherlands’ provinces Limburg (*n* = 3), Noord-Brabant (*n* = 2), Gelderland (*n* = 1), Utrecht (*n* = 3), Noord-Holland (*n* = 3), Drenthe (*n* = 1), and Groningen (*n* = 1). The facilities range from one to 16 living groups per floor, averaging 5.2 living groups. The living groups consist of 6–16 residents, with an average of 8.6 residents. Five different circulation systems are discovered in the floorplan layouts: linear system (*n* = 5), linear system with one or multiple corners (*n* = 3), circular system (residents can walk continuously in the corridor; *n* = 2), a system of two corridors (*n* = 3), and lastly one particular circulation system was noticed: a blending between the corridor and the living room where no separated corridor is used (*n* = 1). Lastly, the principle of the sanitary rooms differs; private individual sanitary room (*n* = 7), a sanitary room shared by two residents (*n* = 3), and a sanitary room shared by more than two residents (*n* = 4).

### Design Criteria

Features and interventions for the floorplan layout design are referred to as design criteria in this research (Marquardt & Schmieg, 2009). In total, 14 design criteria are evaluated, focusing on supporting spatial orientation and wayfinding abilities for seniors with dementia living in an inpatient care facility, such as the sequence of spaces or the provision of visual access (see Table 2).

**Table 2.** Design Criteria Stimulating Wayfinding Abilities for Seniors With Dementia (Each Criterion Is Described With a Number, a Topic, a Description, a Visual, and With Sources).

Criterion	Topic	Definition	Visual	Source(s)
1	Sequence of spaces in the house	The routing inside the house should be in the line of entrance, living room, and individual room of the resident	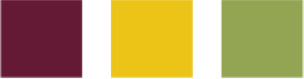	De Vos (2011) and Nilisen & Optiz (2013)
2	Location of the entrance door	The location of the entrance door should not be located at the end of the corridor; it would be better to place it alongside the wall	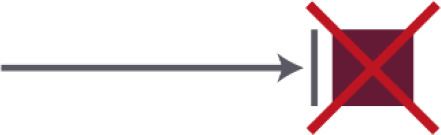	Zeisel et al. (2003)
3	Location of the living room	The location of the living room should be placed at a remarkable place in the building, for example, at the end of the corridor	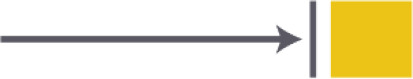	Nilisen & Optiz (2013) and Zeisel et al. (2003)
4	Visual access between entrance and the living room	Provide visual access between the entrance hall and the living room (this increases the orientation skills of the resident, the feeling of home, and a feeling of overview for both the resident and the care professional)	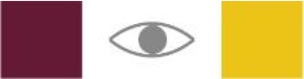	De Vos (2013), Marquardt (2011), Nilisen & Optiz (2013), and Passini et al. (2000)
5	Visual access between the living room and the corridor	Provide visual access between the living room and the corridor	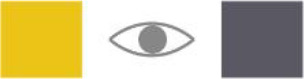	Brawley (1997), Fleming & Purandare (2010), Marquardt (2011), Marquardt & Schmieg (2009), Namazi & Johnson (1991), Nilisen & optiz (2013), and Passini et al. (1998, 2000)
6	Visual access between sanitary and individual room	Provide visual access between the door of the sanitary room from the bed in the individual room	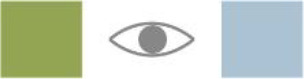	De Vos (2013)
7	Length of the route	Make use of short routes in relation to orientation	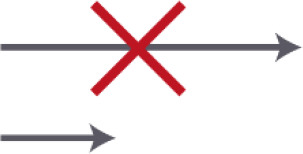	Aedes-Actiz (2018), Marquardt (2011), Nilisen & Optiz (2013), and Van Liempd et al. (2009)
8	Width of the corridor	The corridor should be wide enough for the passage of two persons next to each other and to provide overview	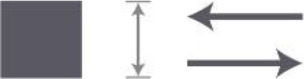	Passini et al. (2000) and Zeisel et al. (2003)
9	Shape of the corridor	Make use of articulated architecture	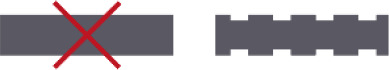	Brawley (1997), Cohen & Weisman (1991), Heeg & Goerlich (2000), Marquardt (2011), Marquardt & Schmieg (2009), Netten (1989), and Passini et al. (1998, 2000)
10	Moments of decision on the route	Decrease the amount of moments of decisions	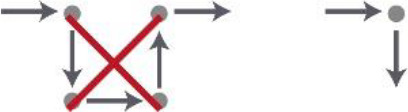	Marquardt (2011)
11	The amount of doors in the corridor	Decrease the amount of doors in the corridor	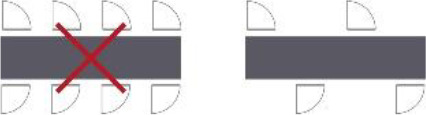	De Vos (2013), Marquardt (2011), Nilisen & Optiz (2013), and Van Liempd et al. (2009)
12	Activity space at the end of the corridor	Locate at the end of the corridor no individual space of the resident, but a space of activity	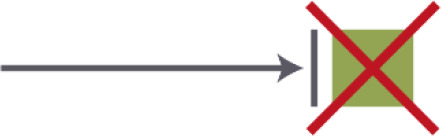	Marquardt & Schmieg (2009), Nilisen & Optiz (2013), Warner (2000), Zeisel (2001), and Zeisel et al. (2003)
13	Entrance of natural daylight	Make use of natural daylight and view outside in the corridor	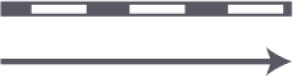	Day et al. (2000), Marquardt (2011), and Zeisel et al. (2003)
14	The amount of doors in the living room	Decrease the amount of doors in the living room	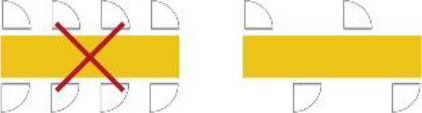	Nilisen & Optiz (2013)
Legend:	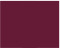 Entrance	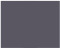 Corridor	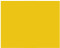 Living room	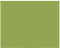 Individual room 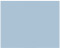 Bathroom	

Dementia is associated with impairments of cognitive spatial skills, resulting in a struggle to produce a mental map of the living environment (Marquardt, 2011). Consequently, visual access to key places is of fundamental importance for the resident. If a senior with dementia can actually see the destination, he is more likely to reach it. Aside from visual access, important spaces for seniors with dementia should be located in remarkable places along the route to be visible and reachable (Zeisel et al., 2003). Visibility and ease of access are also supported by the width of the corridor (Passini et al., 2000).

Another decline in spatial orientation skills due to dementia is the ability to make decisions (Marquardt, 2011). Therefore, it is of importance to limit the decision-making moments along the route to their destination. Articulated architecture can serve as anchor points when decisions on the route need to be made. The length of the route also influences wayfinding skills. If a route is too long, seniors with dementia could forget the destination and get lost. The shorter the route, the easier it will be to reach the destination.

Because of the progressive nature of dementia, it is harder for those living with the condition to adapt to a new environment (Lawton & Simon, 1968). Therefore, it is essential to arrange the sequence of the spaces in a homelike fashion when seniors with dementia have to move to a new living environment: from the public (e.g., entrance hall and collective living room) to private areas (i.e., individual room; De Vos, 2011). These homelike spatial arrangements appear to enhance the chance of reaching destinations. Access to natural daylight in the corridor enhances good vision and seems to provide a better interpretation of the built environment (Marquardt, 2011).

The design criteria are defined by a literature study, including journal articles, conference papers, gray literature, and books. The snowball method (Baarda & de Goede, 2006) has been used with a starting point with the key words “Dementia design” AND “Wayfinding” OR “Spatial orientation” in the Google Scholar engine. The criteria are categorized into two levels: on the spatiofunctional configuration of the building layout (Criteria 1–6) and room characteristics (Criteria 7–14).

### CFA and MCA

The method CFA was applied to analyze the floorplan layouts to provide insights into patterns of spatial relationships (Hoogdalem et al., 1985) and identifies which and how design criteria are implemented into practice. The CFA consists of a process of four steps (van der Voordt et al., 1997): (1) determination of evaluation aspects (the design criteria), (2) measurement of the relevant aspects (see “Analysis” section), (3) evaluation of the outcome (see “Analysis” section), and (4) weighting the importance of the various aspects. The fourth step is usually part of the MCA (Jong & van der Voordt, 2002).

MCA is a method to explore the evaluation of several alternatives Vines et al. (1999). It allows the ranking of a set of alternatives (i.e., optimal design typologies) based on multiple design criteria (Stirling & Davies, 2004; Voogd, 1982). Within the MCA, the performance of each alternative (i.e., floorplan layout of the cases) under each criterion (i.e., design criteria) is evaluated according to its relative importance (Stirling & Davies, 2004). Hypothetically, one design criterion could be of more importance than another, expressed in a different weighting factor. This varying importance influences the evaluation of the design of a floorplan layout. Literature shows that involved stakeholders sometimes magnify a specific aspect—such as affordability or sustainability—within a project evaluation. That specialized aspect is designated with a higher weighting factor. However, in this study, all design criteria are of equal importance because no indications were found in literature to show differences of impact on one of the design criteria, and therefore, the weighting factor for each design criterion is “one.”

For the evaluation of floorplan layouts, standards had to be set for the whole range of design criteria. The floorplan layouts of the cases were first analyzed, and the possibilities per design criterion were written down. These possibilities were compared according to the design criterion, and evaluations were set up (see Table 3). Most of the design criteria were evaluated by a qualitative study, using the scale of − (*bad*), 0 (*neutral*), + (*good*), ++ (*very good*), or +++ (*excellent*). If the design criterion is implemented in a correct manner, this resulted in a positive score (+), and if not, this resulted in a negative score (−). The scores of very good (++) and excellent (+++) are added if the design criterion is implemented even better. For example, an excellent score is given to design Criterion 13 when daylight enters the building from both alongside and the end of the corridor, while a good score means that daylight is only provided at the end of the corridor. The neutral score (0) is applied when the design criterion matches neither positively nor negatively. For example, the Design Criterion 2 prescribes the location of the entrance door alongside the wall, while the assessed neutral scores are applied for the position of the entrance door inside the living room. Although the idea of creating a transitional area from the public domain to a semi-private space (e.g., the living room) is considered to be common knowledge among architects, the analyses of the floor plans confirm that this is not always the case in actual practice. However, the literature indicates that a lack of transitional space is not conducive to seniors’ wayfinding skills. Design Criterion 7 “length of the route,” Criterion 10 “moments of decision,” and Criterion 14 “the number of doors in the living room” were assessed with quantitative dimensions: meters (feet), number of moments of decisions, and number of doors. The highest score of Criteria 1–6, 8, 9, 12, and 13 was evaluated as the best. For Criteria 7, 10, 11, and 15, the lowest score was evaluated as optimal.

**Table 3.** Evaluation Scores Per Design Criterion Stimulating Wayfinding for Seniors With Dementia.

Number of Possibility	Description of Possibility	Score
Criterion 1. Sequence of spaces in the house. The routing inside the house should be in the line of entrance, living room, and individual room of the resident		
Sequence I	Entrance—living room—corridor with individual rooms	**+**
Sequence II	Entrance—(small) corridor—living room/corridor with individual rooms	**+**
Sequence III	Entrance—living room/corridor with individual rooms	**+**
Sequence IV	Entrance—corridor with individual rooms—living room	**−**
Criterion 2. Location of the entrance door. The location of the entrance door should not be located at the end of the corridor; it would be better to place it alongside the wall		
Entrance position I	Alongside the corridor	**++**
Entrance position II	At the end of the corridor in a niche, 90 degrees turned from the end of the corridor	**+**
Entrance position III	In the living room	0
Entrance position IV	Entrance hallway comes out in the living room	0
Entrance position V	At the end of the corridor	**−**
Criterion 3. Location of the living room. The location of the living room should be placed at a remarkable place in the building, for example at the end of the corridor		
Position living room I	At the end of the corridor	**+**
Position living room II	Alongside the entire length of the route	**+**
Position living room III	At the end of the corridor, 90° turned from the end of the corridor	0
Position living room IV	In the middle—alongside—the corridor	0
Position living room V	The entrance hall separates the corridor with the individual rooms and the living room	**−**
Criterion 4. Visual access between entrance and the living room. Provide visual access between the entrance hall and the living room (this increases the orientation skills of the resident, the feeling of home, and a feeling of overview for both the resident and the care professional)		
Visual access I	Yes	**+**
Visual access II	No	**−**
Criterion 5. Visual access between the living room and the corridor. Provide visual access between the living room and the corridor		
Visual access I	Yes	**+**
Visual access II	Yes, softly separated	0
Visual access III	No	**−**
Criterion 6. Visual access between sanitary and individual room. Provide visual access between the door of the sanitary room from the bed in the individual room		
Visual access I and layout I	Yes and the sanitary room attached to the rectangular shaped individual room	**+++**
Visual access I and layout II	Yes and the sanitary room inside the rectangular shaped individual room, and the door of the sanitary room is located at the wide side of the space	**++**
Visual access I and layout III	Yes and the sanitary room inside the rectangular shaped individual room, and the door of the sanitary room is located at the chamfered side of the space	**++**
Visual access I and layout IV	Yes and the sanitary room inside the rectangular shaped individual room, and the door of the sanitary room is located at the smaller side of the space	**+**
Visual access II	No, no individual sanitary room	**−**
Criterion 7. Length of the route. Make use of short routes in relation to orientation		
The longest route from an individual room (furthest away from the living room) to the living room will be measured. This is a quantitative dimension in meters (feet). In the evaluation, the shorter route within the comparison between two cases will be assessed as better		
Criterion 8. Width of the corridor. The corridor should be wide enough for the passage of two persons next to each other and to provide overview		
The smallest passage of the corridor will be measured; quantitative dimension in millimeters (feet). (The width of one wheelchair is 750 mm (2.46 feet), and two wheel chairs next to each other have a width of 1,500 mm)		
Width I	≥1,500 mm (4.92 feet)	**+**
Width II	<1,500 mm (4.92 feet)	**−**
Criterion 9. Shape of the corridor. Make use of articulated architecture		
Shape I	Both sides are differentiated	**++**
Shape II	One side niches, one side differentiated	**++**
Shape III	At both sides niches	**+**
Shape IV	One side straight, one side with niches	**+**
Shape V	One side straight, the other with openings to the living room (formed by interior elements)	**+**
Shape VI	One straight rectangular shape	**−**
Criterion 10. Moments of decision on the route. Decrease the amount of moments of decisions		
The amount of decision moment (which will be explained in the next line) on the longest route from the individual room (furthest away from the living room) to the living room will be calculated. Three types of decision moments can be distinguished: From the individual room the choice: left or right Go around the corner Go through another type of space (e.g., the entrance hall) to enter the living room This is a quantitative dimension in number of moments of decision and in number of moments of decision. In the evaluation, the less number of moments of decision on the route within the comparison between two cases will be assessed as better		
Criterion 11. The amount of doors in the corridor. Decrease the amount of doors in the corridor		
The amount of doors in the corridor which are calculated within this criterion is defined by the following equation. “The total amount of the doors in the corridor” (minus) “The amount of doors of individual rooms in the corridor.” This is a quantitative dimension. In case of two corridors within one case, the corridor with the highest amount of doors in the corridor will be evaluated. In the evaluation, the less amount of doors in the corridor within the comparison between two cases will be assessed as better		
Criterion 12. Activity space at the end of the corridor. Locate at the end of the corridor no individual space of the resident, but a space of activity		
Type of space I	Individual room of a resident	**−**
Type of space II	Individual room of a resident, turned around 90°	**0**
Type of space III	Any other type of space than an individual room of a resident	**+**
Criterion 13. Entrance of natural daylight. Make use of natural daylight and view outside in the corridor		
Daylight I and location I	Yes and alongside and at the end of the corridor	**+++**
Daylight I and location II	Yes and alongside the corridor	**++**
Daylight I and location III	Yes and at the end of the corridor	**+**
Daylight II	No	**−**
Criterion 14. The amount of doors in the living room. Decrease the amount of doors in the living room		
The total amount of doors in the living room will be measured. (This includes also the entrance door to the outside world—either outside in the open air or outside within the larger complex—when this door is situated inside the living room). In the evaluation, the less amount of doors in the living room within the comparison between two cases will be assessed as better		

## Analysis

### Assessment

The aim of the assessment was to gain insights into the implementation of theoretical knowledge into daily practice. It is crucial to determine the reliability of the gathered data during the process of collecting. Due to the use of the floorplan layouts in this study, the provided information is limited to (the setting of) these cases. For example, no data were provided on possible visual access via doors with glass (i.e., Design Criterion 5). In these cases, the door has been considered a closed door. Natural daylight (Design Criterion 13) was also not measured because the height of windows is not included in these floorplan layouts.

An objective evaluation was achieved by involving two peers, both experts in research and design, to exclude personal bias due to possible subjectivity. The author and both peers evaluated each floorplan layout individually, followed by a joint discussion on the results. Table 4 shows the used evaluation matrix.

**Table 4.** Assessment of the floorplans of fourteen cases on design criteria stimulating wayfinding (standardized scores)

Criterion	Cases																	[dimension]	Best
	A. Boswijk	B. De Keyzer	C. De Koekoek	D. De Rietvinck	E. De Schiphorst	F. De Zeven Bronnen	G.A. Heiveld (A)	G.B. Heiveld (B)	H.A. Hogeweyk (A)	H.B. Hogeweyk (B)	I.A. Isselwaerde (A)	I.B. Isselwaerde (B)	J. Juliana	K. Kulturhus Litserborg	L. St. Elisabeth	M. t Loug	N. Wijerode		
Criterion 1 (Sequence of spaces)	+	+	+	-	-	+	+	+	+	+	+	+	-	-	+	+	+	Qualitative	+
Criterion 2 (Location of entrance door)	0	0	0	+	-	++	0	0	-	0	-	-	-	-	-	0	-	Qualitative	+
Criterion 3 (Location of living room)	+	0	+	0	0	0	+	+	-	+	-	-	0	+	0	+	+	Qualitative	+
Criterion 4 (Visual access entrance & living room)	+	+	+	-	-	-	+	+	+	+	+	+	-	-	-	+	-	Qualitative	+
Criterion 5 (Visual access living room & corridor)	0	0	+	+	+	+	+	+	-	+	-	-	+	0	+	+	+	Qualitative	+
Criterion 6 (Visual access sanitary & individual room)	-	+	++	+	++	+++	-	-	-	-	-	-	+++	-	+	+++	+++	Qualitative	+
Criterion 7 (Length of the route)	27	16	18	23	15	41	7	7	17	8	25	7	15	21	20	8	43	[meters and feet]	lowest
Criterion 8 (Width of the corridor)	+	-	+	-	+	+	+	+	+	+	-	-	+	-	+	-	+	Qualitative	+
Criterion 9 (Shape of the corridor)	++	+	+	++	-	+	-	-	+	+	+	+	-	+	-	+	-	Qualitative	+
Criterion 10 (Moments of decision of the route)	1	3	2	2	0	3	2	0	3	2	2	3	2	2	2	0	3	[amount of moments]	lowest
Criterion 11 (Amount of doors in corridor)	8	4	3	7	13	11	3	5	3	4	3	5	4	8	16	6	5	[amounts]	lowest
Criterion 12 (Activity space at the end of the corridor)	+	-	-	0	0	-	-	-	0	-	+	+	+	-	-	+	-	Qualitative	+
Criterion 13 (Natural daylight in corridor)	+	-	+	++	-	+++	-	-	++	-	-	-	-	-	+++	+	++	Qualitative	+
Criterion 14 (Amount of doors in living room)	3	2	2	4	3	1	6	6	5	5	2	2	1	2	2	6	6	[amount]	lowest

### Sensitivity Analysis

Establishing the reliability of the MCA conclusions is a crucial factor, which is determined by the sensitivity analysis. The sensitivity analysis checks whether the ranking of the design layouts provided by the MCA is solid enough and can be conducted by the exclusion of a design criterion (Voogd, 1982). The ranking might be influenced by the design criteria or the weighting factor of the MCA design criteria. Therefore, the ranking of the selected floorplan layouts was calculated once again 14 times when one design criterion was excluded. It turned out that the floorplan layouts of the cases C(J), C(C), and C(M) continued to be in the top three rankings, and C(K) stayed the lowest. This means that the result of the top three rankings is reliable enough.

## Results

### Design Criteria

In order to identify which of the design criteria needs special attention in future developments, each design criterion was evaluated in the floorplan layouts of the cases and described in detail. The different applications of all design criteria were distinguished and assessed (see Table 3).

#### Criterion 1: Sequence of spaces in the house

In most of the cases, the first sequence (entrance—living room—corridor—individual room) is applied, which is considered the correct application of the design criterion. The fourth sequence (entrance—corridor with individual rooms—living room) is applied in four cases, which is negatively assessed.

#### Criterion 2: Location of the entrance door

In almost half of the floorplan layouts, the entrance door of the living group is positioned at the end of the corridor. This position of the entrance door is contradictory to the prescribed design criterion.

#### Criterion 3: Location of the living room

In eight floorplan layouts, the living room is located in a visible and accessible place at the end of the corridor or alongside the entire corridor. One striking feature of the floorplan layout of C(H) with two separate corridors is that, in one corridor, the living room is located at the end of the corridor (assessed as +, because it is an easily visible and accessible place), while, in the other corridor, the entrance hall separates the corridor and the living room (evaluated as −, because the resident has to go through another room in order to reach the living room).

#### Criterion 4: Visual access between the entrance and the living room

In more than half of the floorplan layouts of the cases, a visual access between the living room and the entrance is created. In the C(A) floorplan layout, the entrance and the living room are flowing into each other.

#### Criterion 5: Visual access between the living room and the corridor

In most of the floorplan layouts, visual access between the living room and the corridor is provided. Remarkable is that in C(H)—which has two separate corridors—one corridor has visual access with the living room (judged as +) and the other corridor has no visual access with the living room (judged as −).

#### Criterion 6: Visual access between the sanitary room and individual room

In slightly more than half of the floorplan layouts, visual access was created between the sanitary room and the individual room. This was accomplished through multiple configurations. However, in almost half of the cases, no visual access between these rooms is created. These last have the characteristic that the residents share the bathroom.

#### Criterion 7: Length of the route

The length of the route was shown in meters (feet). The longest route from the individual room to the living room was calculated. The lengths vary between 8 m (26.25 feet) and 43 m (141.08 feet; this is five times longer than the shortest route). The average length is 20 m (65.62 feet), and the mean is 18 m (59.06 feet).

#### Criterion 8: Width of the corridor

In two thirds of the floorplan layouts, the corridor is wide enough for the passage of two persons next to each other. The smallest passage of the corridor was evaluated. The widest corridor is 2.122 m (6.96 feet), and the narrowest corridor has a width of 1.354 m (4.44 feet). Cases narrower than 1.5 m (4.92 feet) are considered suboptimal.

#### Criterion 9: Shape of the corridor

Six different shapes were distinguished, and only one possibility is assessed negatively. However, this option has been observed in more than one third of the cases. The majority has a corridor with one side finishing on a straight line and the other side finishing on niches.

#### Criterion 10: Moments of the decision on the route

Zero to three moments of the decision were distinguished in the floorplan layouts of the cases. In almost half of the cases, the resident needs to make two decisions to go from the individual room to the living room.

#### Criterion 11: The number of doors in the corridor

The number of doors in the corridor—without the doors of individual rooms—varies between three and 16 doors; this is a difference of five times as much. In the corridor, three to five doors are often used.

#### Criterion 12: Activity space at the end of the corridor

This design criterion prescribes an activity space at the end of a corridor instead of an individual room. In half of the cases’ floorplan layouts, the resident’s private room was located at the end of the corridor.

#### Criterion 13: Entrance of natural daylight

In about half of the floorplan layouts of the cases, no access to natural daylight in the form of a window was provided. In two cases, daylight from both the long side of the corridor and the short side of the corridor was provided.

#### Criterion 14: The number of doors in the living room

The number of doors in the living room varies between one and six doors. In six cases, the living room has two doors, and in four cases, it has six doors. The spaces behind the living room doors are the entrance (hall), corridor, outdoor space, storage, nurse office, sanitary, kitchen, and a passage. In most of the floorplan layouts of the cases, the corridor is situated behind one of the living room doors.

### Design Typologies

For determining which design layout is more suitable for people with dementia, the method of MCA was used. The dominance scores were calculated and compared for two alternatives at the time, and thereafter, the overall dominance score was calculated (Voogd, 1982). The overall dominance score determines the ranking of the alternatives (i.e., design layouts). Ranking the cases’ floorplan layouts provides insight into suitable floorplan layouts to meet the needs of seniors with dementia concerning wayfinding.

Based on the shape and position of the circulation system, the floorplan layouts of the analyzed inpatient care facilities are classified into five typologies: (1) a floorplan layout system with one straight corridor, (2) a linear system with one or multiple corners, (3) a floorplan layout with two corridors separated from each other by other functions like the living room, (4) a continuous circular loop to give seniors with dementia a place to wander without encountering obstacles, and (5) the corridor is combined with other functions like the living room or framed by a wall and interior elements (see “Cases” section).

Table 5 shows the ranking of the alternatives and the adherent typological floorplan layout. The majority of the cases are classified in the first type of floorplan layout. The best-ranked cases also belong to this typology. The ranking supports the identification of the most suitable design layout supporting wayfinding skills for seniors with dementia, which is in this study design Typology 1: one straight corridor.

**Table 5.** Ranking of the 14 Cases on 14 Design Criteria Supporting Wayfinding: Overall Score.

Case Title		Floor Plan of the Living Group		Typological Floor Plan	
(J) JULIANA		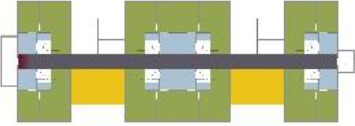		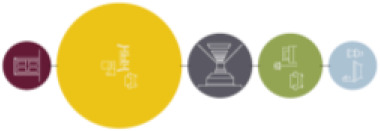	
(C) DE KOEKOEK		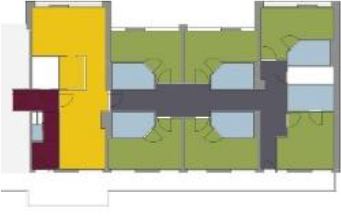		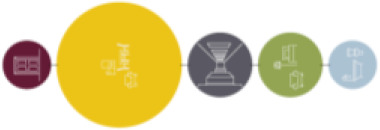	
(M) ‘T LOUG		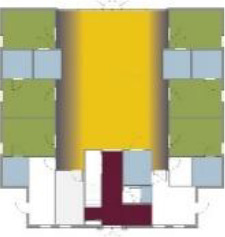		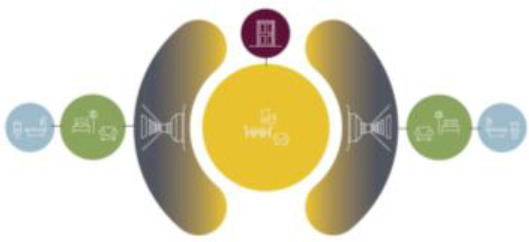	
(F) DE ZEVEN BRONNEN		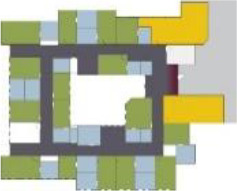		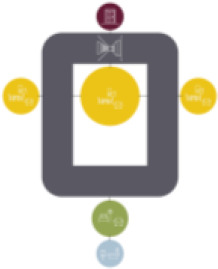	
(D) DE RIETVINCK		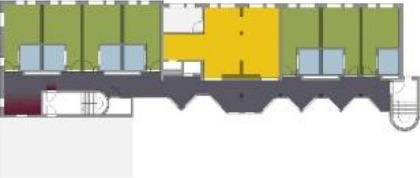		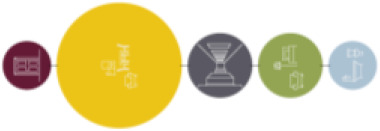	
(A) BOSWIJK		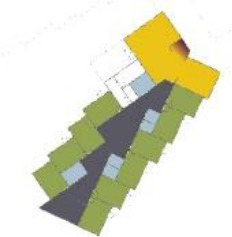		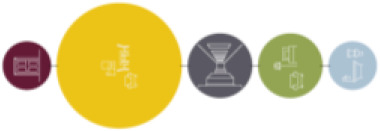	
(E) DE SCHIPHORST		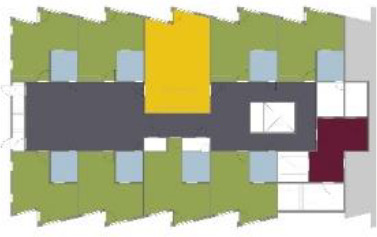		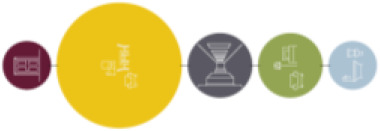	
(H.B) HOGEWEYK (B)		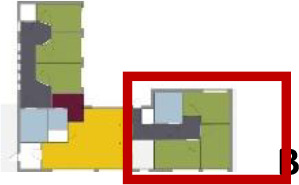		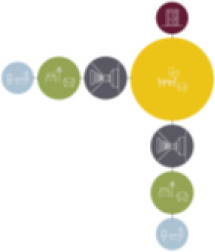	
(L) ST. ELISABETH		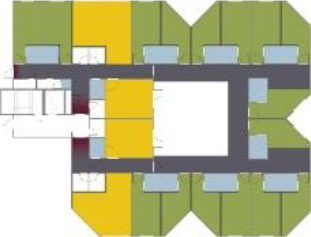		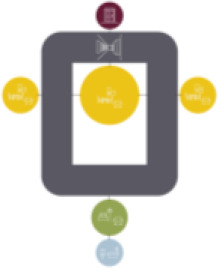	
(H.A) HOGEWEYK (A)		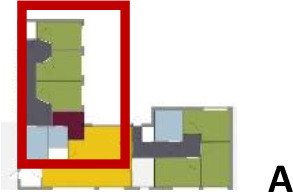		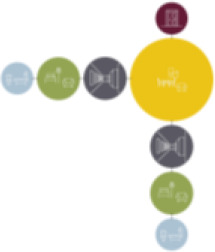	
(G.A) HEIVELD (A)		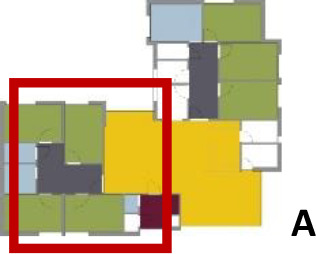		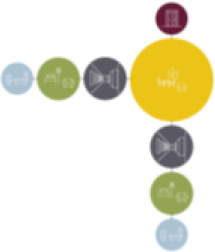	
(G.B) HEIVELD (B)		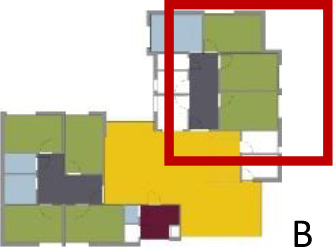		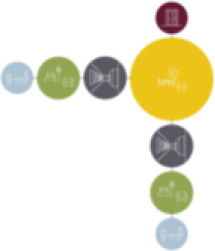	
(I.A) ISSELWAERDE (A)		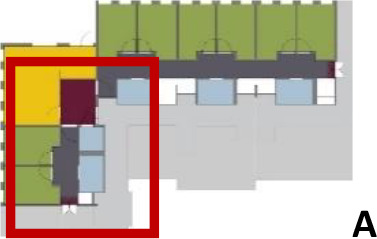		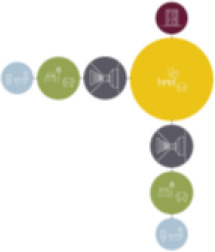	
(I.B) ISSELWAERDE (B)		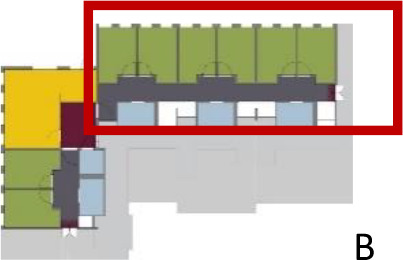		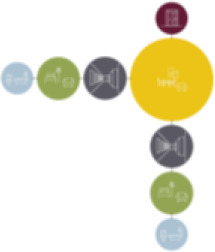	
(N) WIJERODE		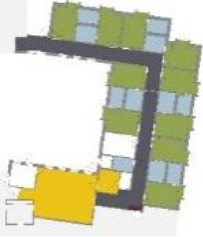		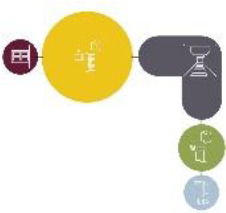	
(B) DE KEYZER		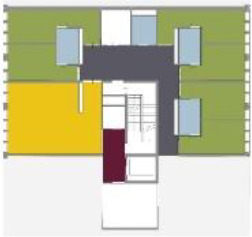		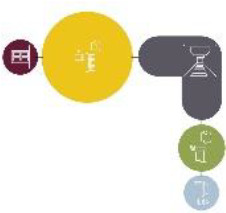	
(K) KULTURHUS LITSERBORG		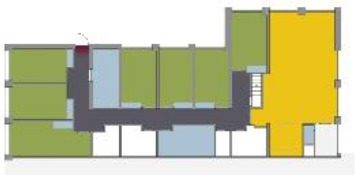		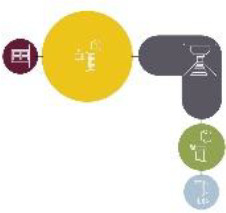	
Legend:	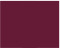 Entrance	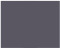 Corridor	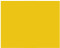 Living room	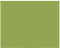 Individual room	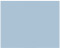 Bathroom

## Discussion and Conclusion

### Discussion

The floorplan layouts of existing care facilities were evaluated on 14 criteria that support wayfinding for seniors with dementia in an inpatient care facility. This study identified which and how design criteria are implemented in current practice. The findings provide insights into factors that require special attention in future developments (see “Results: Design Criteria” section) and identify the optimal design layout to improve the resident’s wayfinding abilities (see “Results: Design Layout” section). This study shows how design criteria and design typologies are interrelated. The design typologies are developed upon the floorplan layouts. The latter ones are evaluated based on the design criteria.

Fourteen cases were evaluated on the spatial implementation of the design criteria. In the evaluation, all design criteria were considered to have equal importance. In literature, no indications were found for any (perceived) impact of a design criterion on wayfinding skills for seniors with dementia. In this research, a CFA and MCA were used to evaluate floor plans based on criteria, but due to the great importance of the user’s perception, a postoccupation evaluation is recommended for future studies.

Further limitations of the study are related to the resource of the assessment: the floorplan layout. The first limitation is that the use of materials, colors, and directional cues (such as arrows or nameplates) could not be assessed, which are elements that support wayfinding skills. Second, using the methodology of assessing in this article and the resources, only quantitative aspects were measured; for example, the sequence of spaces or the position of the entrance door. However, the quality of the applied criteria in the spaces cannot be interpreted by this method. For example, Criterion 13 examined access to daylight, but the amount of daylight was not included. Another limitation is that elements of technology, such as sensors, also could not be assessed. Interactive elements, sensors, and other technology are not visible in floorplan layouts. However, architecture and technology cannot be separated; they are interlinked. The interplay between technological innovation and spatial design has the potential to change the experience of architecture. In that sense, the optimal “experienced design typology” could be different from the optimal design layout based on the criteria in this study, especially for this target group. The floorplan layout of C(M), ranked third in design layout, uses interior elements to create spaces. In architecture, there should be some free space to integrate interior elements and technological elements within the design of the building.

### Conclusions on the Design Criteria (Special Attention in Dementia Architecture)

A part of the study aimed at identifying which design criteria need special attention in future developments. Following the literature and drawing on the results of this empirical study into some cases of inpatient care facilities in the Netherlands, we conclude that in the design of inpatient care facilities for seniors with dementia, special attention needs to be paid to the **configuration of the floorplan layouts**. To stimulate wayfinding, particular attention should be paid to design characteristics that seem to impact significantly **(reducing the number of) decision moments** on different routes. The key design principles can be classified into two main categories:

- Architectural characteristics for creating an effective cognitive map of a space:
  - The shape, width, and length of a corridor should be articulated, spacious (enough room for two passersby), and short.
  - Firstly, the sequence of spaces should allow the resident to adapt more quickly to the (new) living environment. The spatial setting should provide a gentle transition from public to private spaces, in a homelike fashion. Secondly, the living room location should also stand out along the route by situating this room in a clearly visible and accessible place. Thirdly, spaces located at the end of a corridor should have a public function (such as a collective living room) and be accessible for all seniors with dementia. The closed and inaccessible spaces at the end of a corridor often lead to anxiety and agitation among this target group.
- Encouraging the dynamic interaction between the spatial environment and the occupants with dementia to enable their wayfinding skill:
  - Scholars agree that reducing the decision-making moments in a space enhances the possibility of wayfinding. The number of doors, particularly in the living room and along the route, should therefore be limited. The suggested solutions vary from architectural attributes (e.g., centralization, positioning) to rather interior design solutions (e.g., camouflage or highlighting doorways).
  - (Visual) accessibility appears to play an important role in enabling the wayfinding of seniors with dementia. Visual access between the living room, the corridor, and the entrance seems to positively affect their cognitive map. Accessible and visible spaces enhance the decision-making process of the senior. Having clear visual access to the destination will make it easier for seniors with dementia to navigate to those places. The same applies to visual access between the sanitary room and individual room. In almost half of the selected cases, no visual access between those spaces is provided. Characteristics that enhance visibility, such as the smooth entrance of natural daylight, prove to facilitate an adequate interpretation of the environment.

### Conclusions on the Optimal Design Typology

The configuration of the optimal design layout supporting wayfinding skills for seniors with dementia was done based on CFA and MCA. Based on CFA, none of the cases fulfilled all 14 design criteria. All floorplan layouts showed both advantages and disadvantages. The MCA-ranking and the sensitivity analysis provided insights into the best matching floorplan layout for seniors with dementia. The floorplan layouts of C(J), C(C), and C(M) cases are ranked as the top three, while C(K) was ranked the lowest.

The 14 floorplan layouts of the cases are classified into 5 typologies: (1) 1 straight corridor, (2) 1 or multiple corners in the corridor, (3) 2 corridors separated from each other by other functions like the living room, (4) a continuous loop corridor, and (5) a corridor combined with other functions like the living room or framed by a wall and interior elements. The best-ranked floorplan layouts of the cases can be categorized into Typology 1 (C[J] and C[C]) and Typology 4 (C[M]). This is in line with what the literature suggests (Marquardt & Schmieg, 2009). Based on the evaluated 14 design criteria on wayfinding, Typology 3 (two smaller corridors separated from each other) is in no case ranked in the top three. However, if a new facility wants to use this typology for other reasons, the floorplan layout of C(G.B) is often evaluated properly. We recommend conducting a postoccupation evaluation on Typology 3 to verify this result.

Furthermore, it is important to note that floorplan Typologies 1 and 2 basically share the same single linear corridor structure, but the corners appearing in Typology 2 seem to negatively influence the wayfinding skills of seniors with dementia. In the design process, one of the typological floorplan layouts could be chosen as departure point and should be translated toward an actual floorplan layout. The evaluated design criteria in this study could be helpful for this translation in the design process.

## Implications for Practice

- Architects and healthcare professionals should be aware that the physical environment can empower seniors with dementia in finding their way around.
- Architectural guidelines according to the configuration of the floor plan of an inpatient care facility stimulating wayfinding regard a familiar sequence of spaces and special locations for the entrance and living room.
- The corridor is an important connecting element, in which the architect should pay attention to the length, width, shape, moments of decision, and access to daylight in order to stimulate wayfinding.
- Providing visual access between the entrance and living room, between the living room and the corridor, and between the individual room and sanitary room stimulate better wayfinding behavior for seniors with dementia.
- Five typological floor plans are provided in this article. By the translation of one of these typological floor plans toward an actual floor plan, the provided design criteria could help the architect.
